# UNICEF report *Generation 2030 Africa* calls upon investing in and empowering girls and young women

**DOI:** 10.1186/s12978-015-0007-x

**Published:** 2015-03-17

**Authors:** Danzhen You, Lucia Hug, David Anthony

**Affiliations:** Division of Data, Research, and Policy, United Nations Children’s Fund (UNICEF), 3 United Nations Plaza, New York, NY 10017 USA

**Keywords:** Women of reproductive age, Adolescent fertility, Contraceptive prevalence, Unmet need, Africa

## Abstract

UNICEF’s Generation 2030 Africa report released in August 2014, focusing exclusively on Africa, provides an in-depth analysis of child demographic trends. The report highlights the marked increase that Africa population has experienced in the last few decades and the rapid population expansion that is set to continue, with its inhabitants doubling from 1.2 billion to 2.4 billion between 2015 and 2050. A factor driving Africa’s population increase is that the number of women of reproductive age has risen fivefold from 54 million in 1950 to 280 million in 2015 and is set to further increase to 407 million in 2030 and 607 million by 2050. The increasing number of women of reproductive age in Africa will lead to an increasing number of births in Africa even under the assumption of large declines in fertility levels. Adolescent fertility remains high in many African countries and it is estimated that almost one fifth of women in Africa have an unmet need for family planning. The report calls upon investing in and empowering girls and young women and on improving reproductive health of African adolescents.

The African continent has experienced a marked increase in its population over the last few decades. The current population of Africa is five times its size in 1950 and the continent’s rapid population expansion is set to continue with its inhabitants doubling from 1.2 billion to 2.4 billion between 2015 and 2050 and eventually reaching 4.2 billion by 2100 [1]. By using the estimates and projections by the United Nations Population Division, UNICEF’s *Generation 2030 Africa* report takes a look into the demographic shifts in Africa and into the driving forces that have contributed and continue to contribute to the continent’s population increase.

The increasing number of women of reproductive age (15–49 years) in Africa is one of the components driving Africa’s surge in births and children. The number has risen fivefold from 54 million in 1950 to an estimated 280 million in 2015 (Figure 1). This total is projected to increase further to 407 million in 2030 and then to 607 million in 2050, reaching almost 1 billion (991 million) by the end of the century [1]. This population surge will pose substantial challenges for countries in providing services, particularly reproductive health services, antenatal and postnatal care.

**Figure 1:**
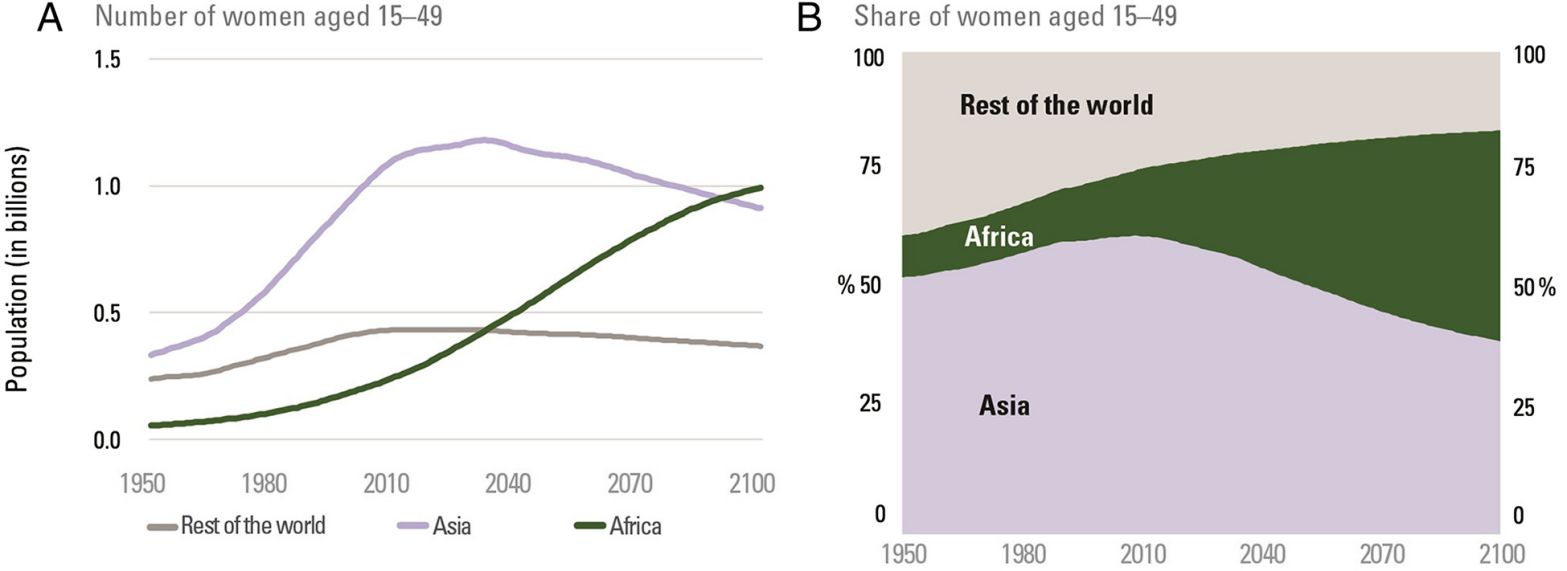
**Africa’s population of women of reproductive age is projected to more than double between 2015 and 2050.** Women of reproductive age by region, 1950–2100 (**A**. Number of women aged 15-49. **B**. Share of women aged 15-49). Source: UNICEF: *Generation 2030 Africa*. New York: UNICEF; 2014.

Another key factor for the African population increase are its continued high fertility levels. Africa’s average fertility level currently stands at 4.5 children per woman of reproductive age, far above the global average of 2.5 children per women. Even though Africa’s average fertility rate is in decline, and has been for decades, its rate of decline is slow and the continent’s fertility rates particularly in Sub-Saharan Africa remains far higher than anywhere else in the world. On current projections, this trend will continue at least until mid-century. Fertility in Africa is projected to drop from around 4.5 children per woman in 2015 to 3.8 in 2030, and to 3.0 by 2050, and further decline to 2.1 children per woman by the end of the century [2].

With the exception of countries with already low levels of fertility rates, significant drops in fertility rates are projected for most African countries over the course of the century — particularly in those countries with the highest rates at the present. But even under the assumption of declines in the fertility rate, the continent’s number of births is not estimated to fall until the 2080s because of the increasing number of women of reproductive age. In fact, the absolute numbers of births are also set to increase massively. On current trends, 700 million babies — slightly under the entire current population of the European continent — will be born in Africa in the next 15 years (2015–2030), with a further 1.1 billion births on the continent between 2031 and 2050.

At the country level current fertility levels on the African continent vary widely from 1.5 children per woman in Mauritius to 7.5 children per women in Niger. In 2015, in 15 African countries women have on average 5 or more children (Figure 2). Often high fertility countries are low income countries and marked by conflict and fragility. Within countries, women in the poorest wealth quintiles tend to have more children than women in the wealthiest quintiles. For example, in Chad, Mali, Niger, Nigeria and the United Republic of Tanzania women in the poorest quintile have on average 2 to 4 children more than women in the wealthiest quintiles [1].

**Figure 2:**
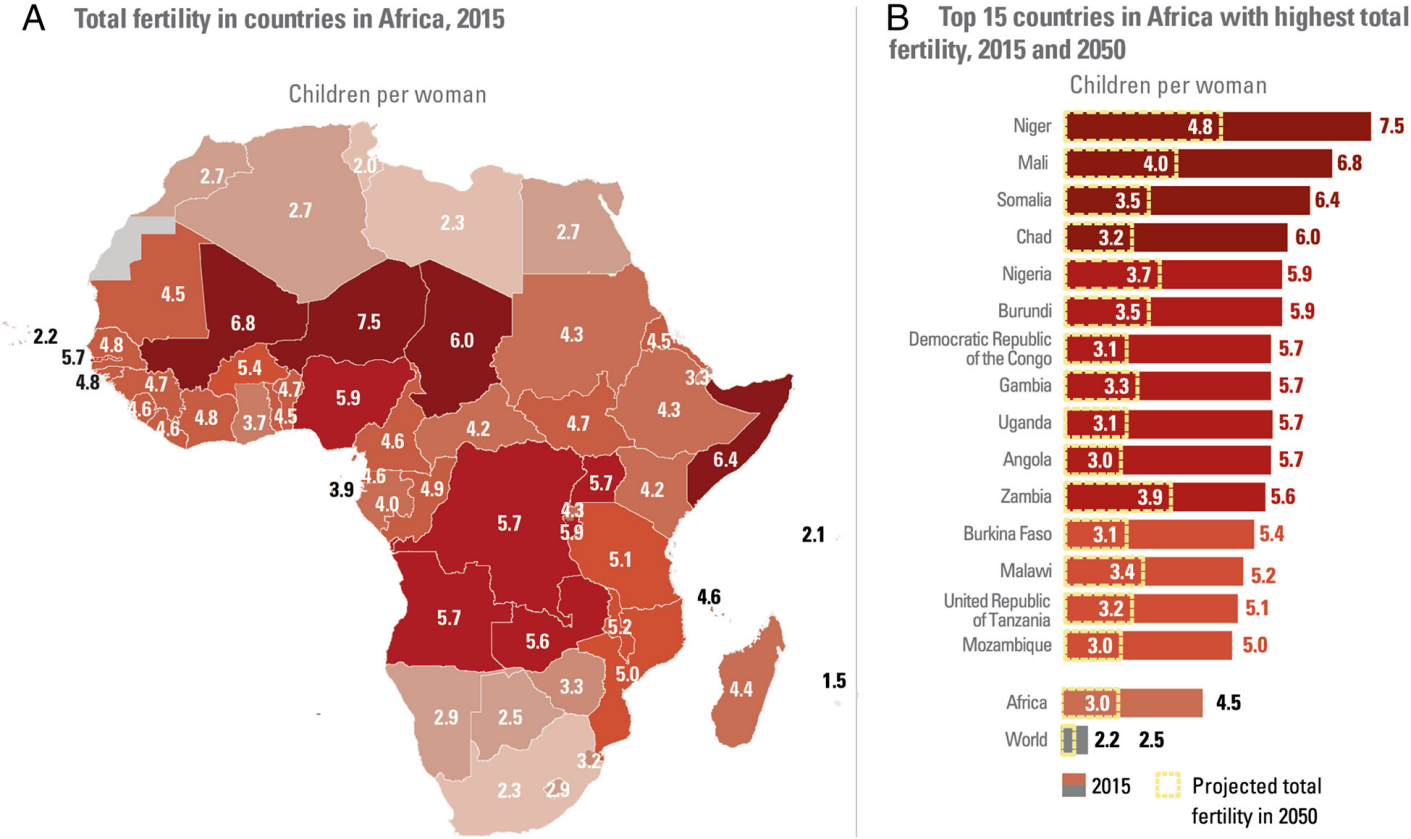
**In 15 African countries, total fertility is at 5 or more children per woman in 2015.** Total fertility in African countries (**A**. Total fertility in countries in African, 2015. **B**. Top 15 countries in Africa with highest total fertility, 2015 and 2050). Source: UNICEF: *Generation 2030 Africa*. New York: UNICEF; 2014.

The report underscores that African women also have among the longest lifetime period for births because of the high rates of adolescent fertility. Africa’s present average adolescent fertility rate is 98 births per 1,000 adolescent girls aged 15–19 years, more than double the worldwide average of 45 births [1] and quadruples the world rate in some of the poorest countries. From 2010 to 2015, 14 per cent of all babies in Africa were born to adolescent girls and women under age 20, compared to 9 per cent globally.

Today worldwide, almost two thirds of women of childbearing age who are in a union are using contraceptive methods. In Africa, this proportion drops to a third of all women. On the continent, 32 African countries have contraceptive prevalence levels below 40 per cent. Half of these countries have an estimated level of contraceptive prevalence below 20 per cent.

Unmet need for family planning reflects the gap between childbearing desires and contraceptive use. Globally, 12 per cent of all women of childbearing age are estimated to have an unmet need for family planning in 2015; for the African continent this proportion rises to 23 per cent [3]. Unmet need for family planning tends to be lowest in countries where contraceptive prevalence is already high (above 60 per cent). In Africa, 38 countries are estimated to have high unmet need levels, ranging from 20 per cent to 35 per cent of all women of reproductive age who are married or in a union (Figure 3). In 28 of them the contraceptive prevalence is below 30 per cent.

**Figure 3:**
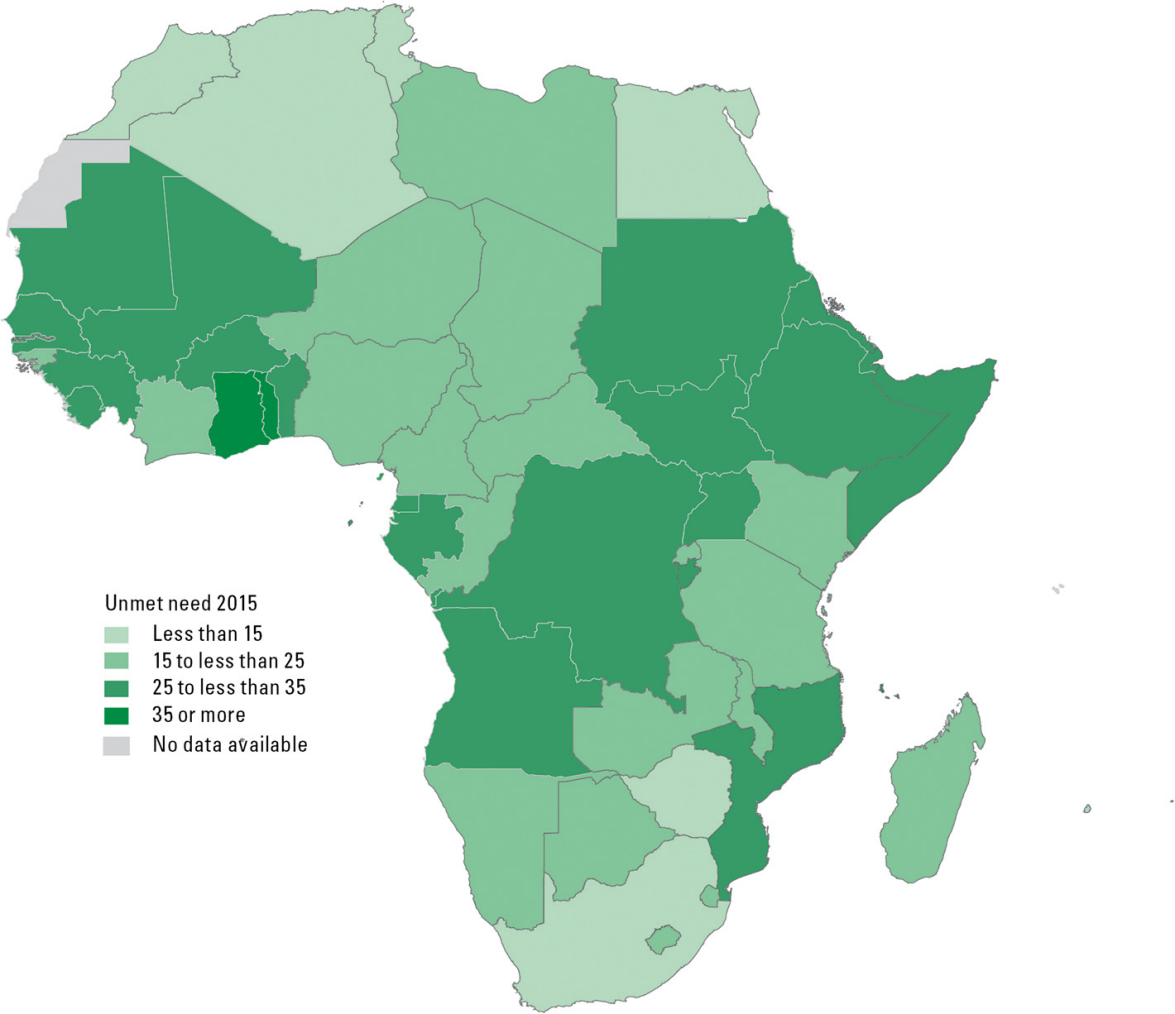
**In about half of the countries in Africa at least a fourth of the women of reproductive age in a union have an unmet need for family planning.** Percentage of married or in-union women aged 15 to 49 who want to stop or delay childbearing but are not using a method of contraception, 2015. Source: UNICEF: *Generation 2030 Africa*. New York: UNICEF; 2014.

High levels of adolescent fertility are associated with elevated rates of unsatisfied demand for reproductive health services, including family planning. In 18 sub-Saharan African countries, more than 50 per cent of adolescent females report unmet need for family planning [4]. A report released by the United Nations Population Division in December 2013 [5] underscored that most countries in sub-Saharan Africa have not seen a notable reduction in unmet need since 1990, in contrast to other regions. However, recent success stories in sub-Saharan Africa (such as Ethiopia, Malawi and Rwanda) show that meeting demand for family planning can be accelerated if reproductive health becomes a higher governmental priority.

A discourse must emerge on how to extend access to greater reproductive health services to Africa’s families — including culturally sensitive reproductive health education and services for women and particularly adolescent girls to reduce the unmet need — in an equitable and socially sensitive fashion that also encourages utilization, is non-discriminatory against any child or woman, and does no harm. Expanded programmes to end child marriage (as defined as a union in which one or both parties are under age 18), which is highly prevalent across the continent, must also be included as part of efforts to invest in and empower girls and young women. Child marriage is a determining factor in sustaining elevated rates of adolescent pregnancy and high lifetime fertility rates for women, and in excluding girls from education. Studies clearly show that educated women delay their first pregnancy, and space their births more widely than women who lack education [4,6,7].

Prioritizing girls’ education — as well as ensuring quality education for all — in Africa will therefore also be among the most powerful measures to build an Africa fit for all. The majority of the world's countries that report high adolescent fertility and low school life expectancy (i.e., the number of years of schooling that a girl pupil can expect to spend from the beginning of primary through secondary school) are in sub-Saharan Africa, where out-of-school rates are also highest [8]. Empowerment of women and girls in Africa must go beyond the statistics, as elsewhere, to the roots of discrimination, marginalization and violence that undermine their rights. Cultural, social, economic and political barriers that perpetuate the disempowerment of women must be urgently addressed.
